# A t-SNE Based Classification Approach to Compositional Microbiome Data

**DOI:** 10.3389/fgene.2020.620143

**Published:** 2020-12-14

**Authors:** Xueli Xu, Zhongming Xie, Zhenyu Yang, Dongfang Li, Ximing Xu

**Affiliations:** ^1^School of Statistics and Data Science, Nankai University, Tianjin, China; ^2^School of Mathematical Sciences, Nankai University, Tianjin, China; ^3^Wuhan National Laboratory for Optoelectronics, Huazhong University of Science and Technology, Wuhan, China; ^4^Key Laboratory for Medical Data Analysis and Statistical Research of Tianjin, Tianjin, China

**Keywords:** microbiome data, dimension reduction, t-SNE, Aitchison distance, classification

## Abstract

As a data-driven dimensionality reduction and visualization tool, t-distributed stochastic neighborhood embedding (t-SNE) has been successfully applied to a variety of fields. In recent years, it has also received increasing attention for classification and regression analysis. This study presented a t-SNE based classification approach for compositional microbiome data, which enabled us to build classifiers and classify new samples in the reduced dimensional space produced by t-SNE. The Aitchison distance was employed to modify the conditional probabilities in t-SNE to account for the compositionality of microbiome data. To classify a new sample, its low-dimensional features were obtained as the weighted mean vector of its nearest neighbors in the training set. Using the low-dimensional features as input, three commonly used machine learning algorithms, logistic regression (LR), support vector machine (SVM), and decision tree (DT) were considered for classification tasks in this study. The proposed approach was applied to two disease-associated microbiome datasets, achieving better classification performance compared with the classifiers built in the original high-dimensional space. The analytic results also showed that t-SNE with Aitchison distance led to improvement of classification accuracy in both datasets. In conclusion, we have developed a t-SNE based classification approach that is suitable for compositional microbiome data and may also serve as a baseline for more complex classification models.

## Introduction

The microbiome in human is involved in a large number of human essential functions, such as metabolism, nutrient intake and energy generation. In recent years, the microbiome has been found to be associated with numerous diseases, and the alterations in that by diet, disease, or environmental factors may impact on human health (Turnbaugh et al., 2006, 2009; Qin et al., 2012; Koeth et al., 2013). The next-generation sequencing technologies make it possible to study the microbiota composition through direct DNA sequencing, replacing classical microorganism study based on isolation and cultivation of specific species. Since the number of the sequence reads is difficult to generate equally for each sample in an experiment, the microbiome data is often required to be converted to the relative abundance for deeper analysis, resulting in compositional microbiome data (McMurdie and Holmes, 2014; Weiss et al., 2017). A single sample can often yield hundreds of millions of short sequencing reads, but for many species they are only observed in a small number of samples, so the microbiome data are typically characterized by high-dimensionality and multivariate sparsity (Li, 2015; Calle, 2019).

To gain a better understanding on the high-dimensional microbiome data, it is essential to reduce the data dimension in such a way that increases interpretability and minimizes information loss simultaneously. Traditional linear dimensionality reduction techniques, such as principal component analysis (PCA) (Hotelling, 1933; Abdi and Williams, 2010), nonnegative matrix factorization (NMF) (Lee and Seung, 1999; Jiang et al., 2012), and classical multidimensional scaling (MDS, also called principle coordinate analysis, PCoA) (Torgerson, 1952; Mugavin, 2008; Gonzalez and Knight, 2012), have difficulty capturing the nonlinear relationships in microbiome data due to their linear nature (Xu et al., 2016; Calle, 2019). In contrast, the nonlinear techniques have advantages in dealing with complex nonlinear datasets (Maaten et al., 2009). Among nonlinear dimension reduction algorithms, t-distributed stochastic neighbor embedding (t-SNE), developed by van der Maaten and Hinton (2008), has recently received increasing attention and has been applied to dimension reduction and visualization of microbiome data (Kostic et al., 2015), single-cell RNA-sequencing data (Linderman et al., 2019), bird songs (Deny et al., 2016), computational fluid dynamics (Wu et al., 2017), genomic data (Li et al., 2017), remote sensing images (Song et al., 2018) and many other application fields.

The t-SNE algorithm could efficiently project complex data sets onto a 2D or 3D plane, while the local structure of the data in the original high-dimensional space is preserved as much as possible. However, the t-SNE method does not provide a built-in way to map new data points to the corresponding low-dimensional representation, and hence it is hardly utilized for classification or regression tasks (Maaten, 2009). Some studies have attempted to cope with this out-of-sample extension problem by using neural networks for feature extraction and then perform classification on the mapped low-dimensional space from t-SNE (Maaten, 2009; Oliveira et al., 2018). However, there is little research on the application of t-SNE to the classification of microbiome data, which may be due to the unique characteristics of microbiome data, such as compositionality and relatively small sample size in many cases, limiting the performance of existing methods on such type of data.

In this article, we explore the potential of t-SNE for the classification of microbiome data, and propose a t-SNE based classification approach, which enables us to build classifiers and classify new samples in the reduced dimensional space. In our t-SNE algorithm, Aitchison distance, introduced by Aitchison (1986), is used to calculate the conditional probabilities for compositional microbiome data. To classify a new sample, its low-dimensional features are first obtained as the weighted mean vector of its nearest neighbors. Using the low-dimensional features as input, three commonly used methods-logistic regression (LR), support vector machine (SVM), and decision tree (DT) are then applied for classification in this study.

## Methods

### t-Distributed Stochastic Neighbor Embedding

For a given set of *p*-dimensional samples ***s***_1_, ***s***_2_, ⋯, ***s***_*N*_ the similarity between sample ***s***_*j*_ and sample ***s***_*i*_ is represented by the conditional probability *p*_*j*|*i*_, *i*, *j* = 1, 2, ⋯, *N*. For nearby samples, *p*_*j|i*_ is relatively high, whereas for widely separated samples, *p*_*j|i*_ will be almost zero. The conditional probability *p*_*j|i*_ is given as

$$p_{j|i}=\frac{\text{exp}\left(-\frac{d^{2}\left(s_{i},s_{j}\right)}{2\sigma_{i}^{2}}\right)}{\sum_{k\neq i}\text{exp}\left(-\frac{d^{2}\left(s_{i},s_{k}\right)}{2\sigma_{i}^{2}}\right)}\;\text{for}i\neq j,\text{and}p_{i|i}=0,$$

Where *d*^2^ (***s***_*i*_, ***s***_*j*_) is the square of the Euclidean distance between sample ***s***_*i*_ and sample ***s***_*j*_ and $\sigma_{i}^{2}$ is the variance of the Gaussian distribution that is centered on sample ***s***_*i*_.

To circumvent the outlier problem, the symmetrized conditional probability between sample ***s***_*i*_ and sample ***s***_*j*_ is recommended,

$$p_{\mathrm{ij}}=\frac{p_{j|i}+p_{i|j}}{2N}\;\text{for}i\neq j,\text{and}p_{\mathrm{ii}}=0.$$

the next step, t-SNE attempts to learn a *d*-dimensional map ***z***_1_, ***z***_2_, ⋯, ***z***_*N*_ (*d* < *p*) that reflects the similarities *p*_*ij*_ between two samples ***z***_*i*_ and ***z***_*j*_ in the reduced dimensional space. The measure of pairwise similarities in the reduced dimensional space uses a student t-distribution rather than a Gaussian distribution to alleviate crowding problem, defined as

$$q_{\mathrm{ij}}=\frac{{\left(1+d^{2}\left(z_{i},z_{j}\right)\right)}^{-1}}{\sum_{k\neq l}{\left(1+d^{2}\left(z_{k},z_{l}\right)\right)}^{-1}}\;\text{for}i\neq j,\text{and}q_{\mathrm{ii}}=0,$$

where *q*_*ij*_ represents the local structure of the data points in the reduced dimensional space. To select the map points so that the two similarity matrices, *P* and *Q*, are as similar as possible, the location of the sample ***z***_*i*_ is determined by minimizing the Kullback–Leibler divergence (Kullback and Leibler, 1951) between the low-dimensional and high-dimensional similarity distributions *Q* and *P*,

$$\mathrm{KL}\left(P∥Q\right)=\sum_{i\neq j}p_{\mathrm{ij}}\log\frac{p_{\mathrm{ij}}}{q_{\mathrm{ij}}},$$

using a gradient-descent method. It is worth noting that the gradient descent is an iterative optimization algorithm, and thereby the iterative number (*iter*) should be optimized. The t-SNE method has another main parameter, the perplexity (*per*), which governs the variance of the Gaussian $\sigma_{i}^{2}$ appearing in the conditional probability *p*_*j|i*_. For a detailed introduction of the t-SNE algorithm, see the literature (Maaten and Hinton, 2008).

### t-SNE Based Classification for Compositional Data

**FIGURE 1:**
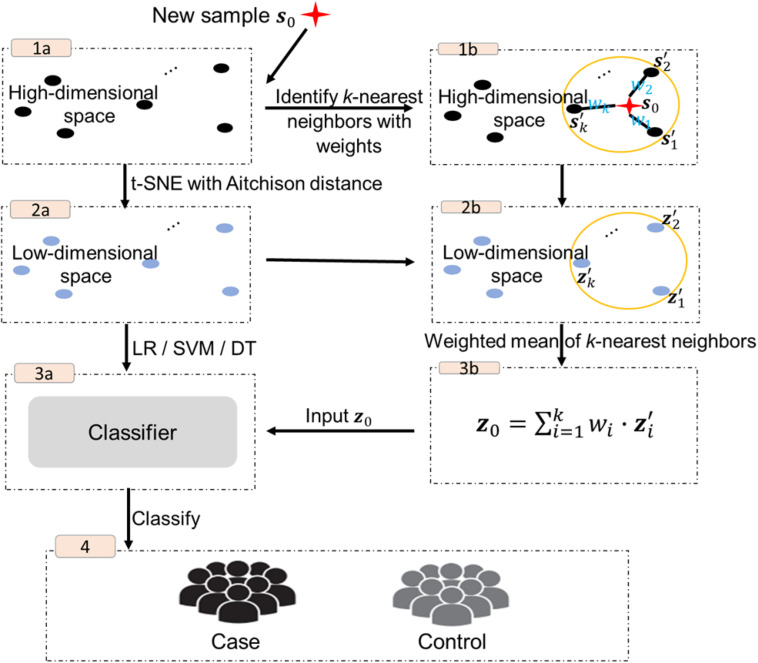
Overview of the procedure of our approach.

The procedure of the proposed approach is shown in Figure 1, including two main parts, the implementation of t-SNE with Aitchison distance and the out-of-sample extension.

#### t-SNE With Aitchison Distance

The sample space of compositional data is simplex (Calle, 2019). Three important conditions should be fulfilled for a proper analysis of compositions: permutation invariance, scale invariance and sub-compositional coherence (Aitchison, 1986). In fact, Euclidean distance cannot meet the principles of scale invariance and sub-compositional coherence, which may lead to spurious correlations among the abundances of the different taxa (Aitchison, 1986; Li, 2015; Calle, 2019). On the other hand, Aitchison distance, which has been proved to satisfy all these criteria, was often deemed to be a solution to most problems related to compositional data (Aitchison, 1992; Calle, 2019).

The Aitchison distance *d*_*a*_ between two *p*-dimensional vectors **x** = (*x*_1_, *x*_2_, ⋯, *x*_*p*_) and **y** = (*y*_1_, *y*_2_, ⋯, *y*_*p*_), is defined as,

$$d_{a}\left(x,y\right)={\left[\sum_{i=1}^{p}{\left(\ln\frac{x_{i}}{\text{g}\left(x\right)}-\ln\frac{y_{i}}{\text{g}\left(y\right)}\right)}^{2}\right]}^{1/2},$$

where g(⋅) denotes the geometric mean. The conditional probability *p*_*j|i*_ in t-SNE is substituted by *p*_*j*|*i*_(*i*, *j* = 1, 2, ⋯, *N*, *j* = 1, 2, ⋯, *N*) calculated as,

$$p_{j|i,a}=\frac{\text{exp}\left(-\frac{d_{a}^{2}\left(s_{i},s_{j}\right)}{2\sigma_{i}^{2}}\right)}{\sum_{k\neq i}\text{exp}\left(-\frac{d_{a}^{2}\left(s_{i},s_{k}\right)}{2\sigma_{i}^{2}}\right)},\text{for}i\neq j,\text{and}p_{i|i,a}=0.$$

The high-dimensional feature vectors of the original set are submitted to t-SNE with Aitchison distance for data dimensionality reduction (step 1a–2a in Figure 1), and the corresponding reduced dimensional data ***z***_1_, ***z***_2_, ⋯, ***z***_*N*_ in *ℝ*^*d*^ are used to build classifiers. In this study, we consider three widely used classification algorithms: logistic regression (LR), support vector machine (SVM) or decision tree (DT) (step 3a in Figure 1).

#### Out-of-Sample Extension

Given a new *p*-dimensional sample ***s***_0_ in *S*^*P*^, its *k* nearest neighbors in the original data are first identified based on the conditional probabilities between ***s***_0_ and the original set of samples (step 1b in Figure 1),

$${\mathrm{sp}}_{0,i}=\frac{\text{exp}\left(-d_{a}^{2}\left({\text{s}}_{0},{\text{s}}_{i}\right)/2\sigma_{i}^{2}\right)}{\sum_{h\neq i}\text{exp}\left(-d_{a}^{2}\left({\text{s}}_{i},{\text{s}}_{h}\right)/2\sigma_{i}^{2}\right)},i=1,2,\cdots ,N.$$

Let *sp*_0,(1)_ > *sp*_0,(2)_ > ⋯ > *sp*_0,(*k*)_ > ⋯ > *sp*_0,(*N*)_ denote the set of *N* ordered probabilities, and $z_{1}^{\prime },z_{2}^{\prime },\cdots ,z_{k}^{\prime }$ denote the low-dimensional representations of the original data with the largest *k* conditional probabilities, which will be labeled as the *k* nearest neighbors of ***s***_0_ step 2b in Figure 1). Then the low-dimensional representation of ***s***_0_ step 3b in Figure 1) is given by

$$z_{0}=\sum_{i=1}^{k}w_{i}\cdot z_{i}^{\prime },$$

where $w_{i}=\frac{sp_{0,\left(i\right)}}{\sum_{i=1}^{k}sp_{0,\left(i\right)}}$ denotes the weight of $z_{i}^{\prime }$.

In the final step ***z***_0_, used as an input to the classifier for prediction (step 4 in Figure 1).

### Selection of Optimal Parameters

In t-SNE, there are several parameters that need to be tuned for good performance, such as the perplexity *per*, which is defined as a smooth measure of the effective number of neighbors. It has been suggested that a typical value for this parameter is between 5 and 50 (Maaten and Hinton, 2008). In practice, proper tuning of *per* requires users to understand the inner working of the t-SNE method as well as to have hands-on experience. In our study, the tuning of parameters can be achieved based on the performance of classifications. In particular, a grid search with fivefold cross-validation is used to tune the parameters, including the perplexity *per*, maximum number of iterations *iter*, output dimensionality *dim*, and number of neighbors *k*. The optimal combination of parameters is selected via maximizing mean cross-validation accuracy.

In addition, for the parameters of the three classification algorithms applied in the reduced dimensional space, LR is trained by tuning the lambda based on minimum mean cross-validated error. In the SVM model, the radial basis kernel is used. The two tuning parameters (gamma and cost) are chosen by minimizing the mean cross-validation error as the best combination for seven values from 10^–6^ to 10^1^ for the gamma and five values from 1 to 5 for the cost. For DT, the optimized decision tree is obtained by evaluating the cross-validated error using a grid search method and then determining the best set of hyperparameters, including min split, min bucket, max depth, and complexity. The rest of the unmentioned parameters uses the default setting in the R package.

### Model Evaluation

To compare the performance of different model settings, we use the area under the receiver operating characteristic curve (AUC) which represents the trade-off between the true positive rate (specificity) vs. the false positive rate (1-sensitivity), the commonly used classification metric-accuracy (ACC) which were defined by the ratio of the samples correctly classified to the total samples, as well as two other criteria capable of overcoming the class imbalance issue, the area under the precision-recall curve (AUPR) which represents the trade-off between the precision vs. the recall, and the normalized Matthews correlation coefficient (nMCC) which projects the original range of MCC [-1, 1] into the interval [0, 1] (Matthews, 1975; Chicco and Jurman, 2020),

$$\mathrm{ACC}=\frac{\mathrm{TP}+\mathrm{TN}}{\mathrm{TP}+\mathrm{FP}+\mathrm{TN}+\mathrm{FN}},$$

$$\mathrm{nMCC}=\frac{\mathrm{MCC}+1}{2},$$

where $\text{MCC}=\frac{\mathrm{TP}\times \mathrm{TN}-\mathrm{FP}\times \mathrm{FN}}{\sqrt{\left(\mathrm{TP}+\mathrm{FP}\right)\left(\mathrm{TP}+\mathrm{FN}\right)\left(\mathrm{TN}+\mathrm{FP}\right)\left(\mathrm{TN}+\mathrm{FN}\right)}}$,

TP, FP, TN, and FN denote true positive, false positive, true negative, and false negative, respectively. For the given data, we randomly split the data to the training and test sets in 80/20 ratio. All of the training samples were randomly divided into five sets, four of which were employed for constructing the classification model, and the remaining one as the validation set was used to validate the model for obtaining the optimal parameters. The generalization performance results were reported by ACC, nMCC, AUC, and AUPR, which were measured on test data that was held out during the training of t-SNE with Aitchison distance or t-SNE with Euclidean distance.

### Implementation of the Proposed Approach

The proposed approach was implemented on R software (version 4.0.2), where t-SNE was performed using the R package tsne, LR was implemented using the R package glmnet, SVM was executed using the R package e1071, and DT was implemented using the R package rpart. In addition, both AUC and AUPR were calculated using the R package PRROC, and MCC was calculated using the R package mltools. The R code could be found at https://github.com/Xuxl2020/t-SNE-classifier.

### Application to Microbiome Data

The proposed approach was performed on two microbiome datasets from diverse body sites: (1) the Mycoplasma pneumoniae (MP) infection data (Zhou et al., 2020) and (2) the idiopathic central precocious puberty (ICPP) data (Dong et al., 2020). The MP infection data was oropharyngeal (OP) microbiota derived from the MP infection study on 99 Chinese children, including 40 patients (diagnosed as MP infection, Case group) and 59 age-matched healthy children (Control group). The ICPP data was fecal microbiota from 25 girls (Case group) with idiopathic central precocious puberty and 23 healthy girls (Control group) in China. All microbiota data were generated from the Miseq platform by sequencing the V3-V4 hypervariable region of microbial 16S rDNA and were annotated with the RDP database and then calculating relative abundance for each sample in the genus taxonomic level. Both data were the compositional data. The MP infection and ICPP data contained 728 and 146 features (genus), respectively.

## Results

### Data Visualizations With t-SNE

**FIGURE 2:**
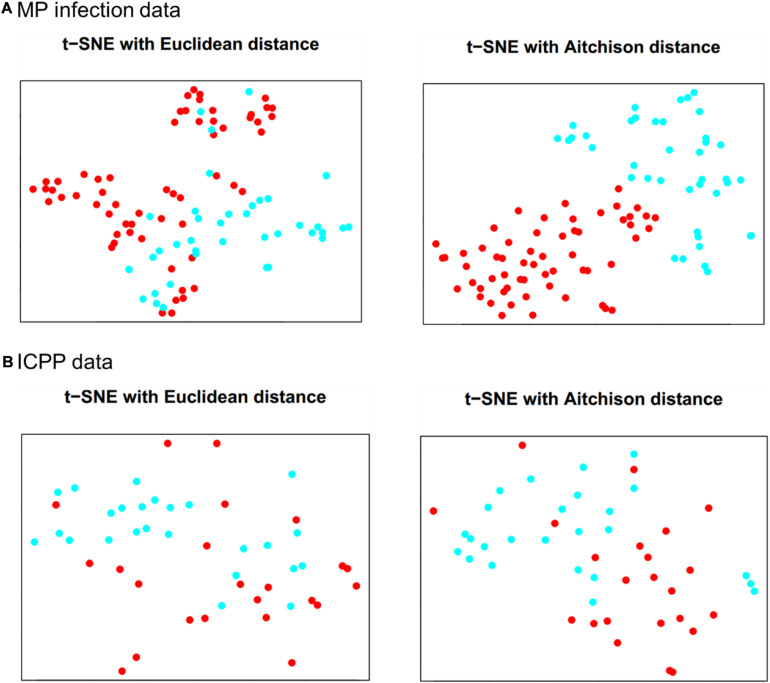
Data visualizations in 2D by t-SNE for **(A)** MP infection data and **(B)** ICPP data. Color: blue for case group and red for control group.

The results of t-SNE 2D map for MP infection data (*per* = 30, *iter* = 2,000) and ICPP data (*per* = 15, *iter* = 2,000) are illustrated in Figure 2. For MP infection data (Figure 2A), t-SNE with Aitchison distance constructs a map in which the separation between the case and control groups is almost perfect. In contrast, t-SNE with Euclidean distance produces a map in which there is no clear boundary between different groups. For ICPP data (Figure 2B), the map produced by t-SNE with Aitchison distance contains a few points that are clustered with the wrong group, probably due to more complex composition and more distinct individual differences in gut microbiota. Again, none of the groups are clearly separated in the t-SNE with Euclidean distance map. The computation time (seconds) of t-SNE with Aitchison distance and t-SNE with Euclidean distance for both microbiome datasets was also provided in Supplementary Table 1.

### Impact of Output Dimensionality on Classification Performance

**FIGURE 3:**
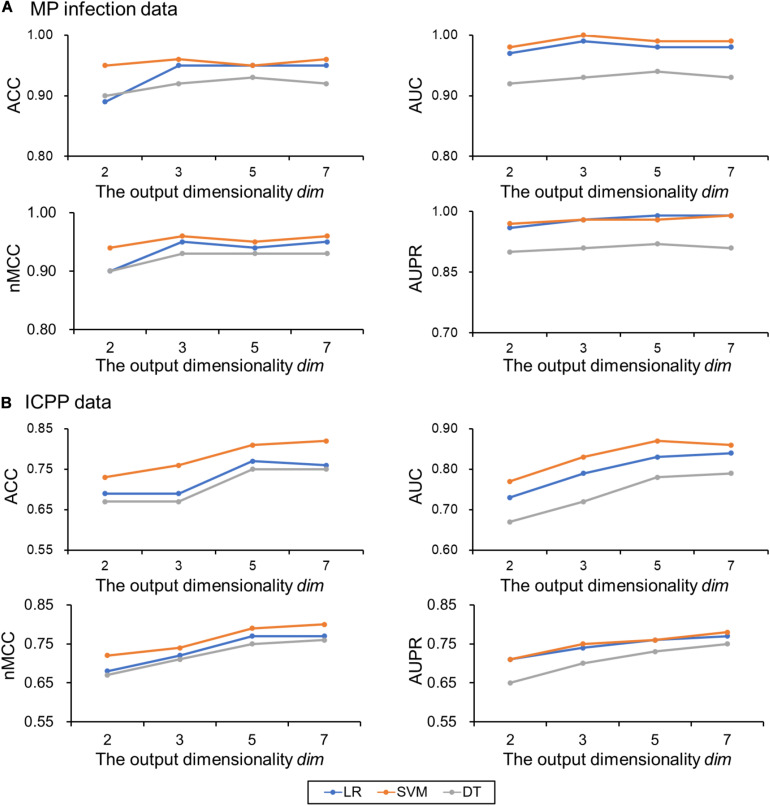
Classification performances on the test data change with the output dimensionality *dim*. **(A)** MP infection data and **(B)** ICPP data. Color: blue for logistic regression (LR), orange for support vector machine (SVM), and gray for decision tree (DT). ACC, the classification accuracy; AUC, the area under the receiver operating characteristic curve; nMCC, the normalized Matthews correlation coefficient; AUPR, the area under the precision-recall curve.

For MP infection data, the optimal parameters selected are: the perplexity *per* = 30, maximum number of iterations *iter* = 2,000, and number of neighbors *k* = 7. To examine the impact of the output dimensionality on the classification performance, the results of the proposed approach (using Aitchison distance) for *dim* = 2, 3, 5, and 7 were presented in Figure 3A. ACCs, AUCs, nMCCs, and AUPRs were relatively small at *dim* = 2. When *dim* = 3, the ACCs were increasing to 0.95, 0.96 and 0.92 for LR, SVM, and DT, respectively, the AUCs were increasing to 0.99, 1.00, and 0.93 for LR, SVM, and DT, respectively, the nMCCs were increasing to 0.95, 0.96, and 0.93 for LR, SVM, and DT, respectively, and the AUPRs were increasing to 0.98, 0.98, and 0.91 for LR, SVM, and DT, respectively, which were similar to the values at *dim* = 5 and 7.

For ICPP data, the optimal parameters selected are: the perplexity *per* = 15, maximum number of iterations *iter* = 2,000, and number of neighbors *k* = 7. The results of the proposed approach (using Aitchison distance) for *dim* = 2, 3, 5, and 7 were presented in Figure 3B. ACCs, AUCs, MCCs, and AUPRs were relatively small at *dim* = 2 and 3. When *dim* = 5, the ACCs were increasing to 0.77, 0.81, and 0.75 for LR, SVM, and DT, respectively, the AUCs were increasing to 0.83, 0.87, and 0.78 for LR, SVM, and DT, respectively, the nMCCs were increasing to 0.77, 0.79, and 0.75 for LR, SVM, and DT, respectively, and the AUPRs were increasing to 0.76, 0.76, and 0.73 for LR, SVM, and DT, respectively, which were similar to the values at *dim* = 7. The detailed results for different output dimensions were summarized in Table 1.

**TABLE 1:** Performance of classification models on the test set.

			MP infection				ICPP			
		*dim* =	2	3	5	7	2	3	5	7
ACC	ED	LR	0.72	0.87	0.88	0.87	0.55	0.59	0.68	0.67
		SVM	0.81	0.87	0.85	0.85	0.61	0.63	0.64	0.64
		DT	0.75	0.84	0.83	0.84	0.60	0.57	0.63	0.64
	AD	LR	0.89	0.95	0.95	0.95	0.69	0.69	0.77	0.76
		SVM	0.95	0.96	0.95	0.96	0.73	0.76	0.81	0.82
		DT	0.90	0.92	0.93	0.92	0.67	0.67	0.75	0.75
nMCC	ED	LR	0.72	0.87	0.87	0.87	0.60	0.61	0.71	0.72
		SVM	0.78	0.87	0.86	0.86	0.63	0.64	0.72	0.72
		DT	0.73	0.82	0.82	0.82	0.62	0.60	0.72	0.71
	AD	LR	0.90	0.95	0.94	0.95	0.68	0.72	0.77	0.77
		SVM	0.94	0.96	0.95	0.96	0.72	0.74	0.79	0.80
		DT	0.90	0.93	0.93	0.93	0.67	0.71	0.75	0.76
AUC	ED	LR	0.77	0.91	0.91	0.90	0.63	0.72	0.77	0.75
		SVM	0.85	0.90	0.89	0.90	0.70	0.74	0.75	0.75
		DT	0.75	0.85	0.82	0.81	0.66	0.68	0.74	0.74
	AD	LR	0.97	0.99	0.98	0.98	0.73	0.79	0.83	0.84
		SVM	0.98	1.00	0.99	0.99	0.77	0.83	0.87	0.86
		DT	0.92	0.93	0.94	0.93	0.67	0.72	0.78	0.79
AUPR	ED	LR	0.72	0.86	0.88	0.90	0.66	0.71	0.74	0.74
		SVM	0.72	0.85	0.86	0.87	0.66	0.71	0.74	0.75
		DT	0.69	0.81	0.82	0.81	0.64	0.69	0.71	0.74
	AD	LR	0.96	0.98	0.99	0.99	0.71	0.74	0.76	0.77
		SVM	0.97	0.98	0.98	0.99	0.71	0.75	0.76	0.78
		DT	0.90	0.91	0.92	0.91	0.65	0.70	0.73	0.75

*ACC, the classification accuracy; nMCC, the normalized Matthews correlation coefficient; AUC, the area under the receiver operating characteristic curve; AUPR, the area under the precision-recall curve; AD, the models using Aitchison distance; ED, the models using Euclidean distance; LR, logistic regression; SVM, support vector machine; DT, decision tree.*

### Impact of Different Distance Measures on Classification Performance

For MP infection data with *dim* = 3, compared to the results using Euclidean distance, the proposed approach using Aitchison distance increased the ACC by 9% for LR, 10% for SVM, and 10% for DT, respectively, increased the nMCC by 9% for LR, 10% for SVM, and 13% for DT, respectively, increased the AUC by 9% for LR, 11% for SVM, and 9% for DT, respectively, and increased the AUPR by 14% for LR, 16% for SVM, and 14% for DT, respectively. For MP infection data with *dim* = 5, compared to the results using Euclidean distance, the proposed approach using Aitchison distance increased the ACC by 13% for LR, 27% for SVM, and 19% for DT, respectively, increased the nMCC by 8% for LR, 10% for SVM, and 4% for DT, respectively, increased the AUC by 8% for LR, 16% for SVM, and 5% for DT, respectively, and increased the AUPR by 3% for LR, 3% for SVM, and 3% for DT, respectively. The detailed results for the comparisons were summarized in Supplementary Table 2.

### Classification Performance in Original and Reduced Dimension Space

**FIGURE 4:**
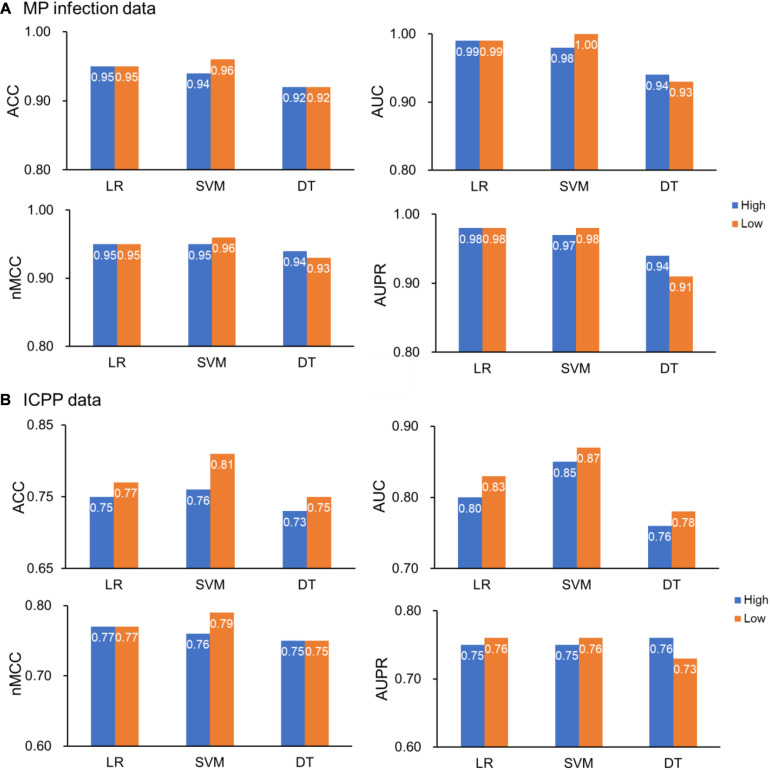
Classification performances in original and reduced dimensional space. **(A)** MP infection data and **(B)** ICPP data. Color: blue (High) for original high-dimensional data and orange (Low) for low-dimensional data. ACC, the classification accuracy; AUC, the area under the receiver operating characteristic curve; nMCC, the normalized Matthews correlation coefficient; AUPR, the area under the precision-recall curve. Note: in the reduced dimensional space, *dim* = 3 and *dim* = 5 for MP infection data and ICPP data, respectively.

To compare the classification performances of the classifiers built in original and reduced dimensional space, we also used the three algorithms (LR, SVM, and DT) to build classifiers in the original dimensional space. For MP infection data (99 samples with 728 features), the ACCs were 0.95, 0.94, and 0.92 for LR, SVM, and DT, respectively, the nMCCs were 0.95, 0.95, and 0.94 for LR, SVM, and DT, respectively, the AUCs were 0.99, 0.98, and 0.94 for LR, SVM, and DT, respectively, and the AUPRs were 0.98, 0.97, and 0.94 for LR, SVM, and DT, respectively (Figure 4A). For ICPP data (48 samples with 146 features), the ACCs were 0.75, 0.76, and 0.73 for LR, SVM, and DT, respectively, the nMCCs were 0.77, 0.76, and 0.75 for LR, SVM, and DT, respectively, the AUCs were 0.80, 0.85, and 0.76 for LR, SVM, and DT, respectively, and the AUPRs were 0.75, 0.75, and 0.76 for LR, SVM, and DT (Figure 4B). In comparison with the results of the proposed approach (Figure 4 and Table 1), we found that the application of dimensionality reduction technique, t-SNE with Aitchison distance, resulted in no reduction in classification accuracy.

## Discussion

In this work, we proposed a classification approach based on t-SNE, taking into account the compositional characteristic of microbiome data. The application of the proposed approach was illustrated on two disease-associated microbiome datasets, and demonstrated good classification performance on both datasets. Although we focused on the classification tasks, the proposed approach could be also used for regression analysis in the reduced dimensional space by t-SNE.

In both microbiome datasets, using the Aitchison distance to calculate the conditional probabilities in t-SNE made the case and control groups appear more clearly separated, compared to the t-SNE map with Euclidean distance (Figure 2), whereas the use of Aitchison distance did not increase the computation time of t-SNE, compared to the use of Euclidean distance (Supplementary Table 1). The classification performance was also improved for the proposed approach by using Aitchison distance (Table 1 and Supplementary Table 2). This was probably because Aitchison distance satisfies the principles of scale invariance and sub-compositional coherence, and hence is more suitable for compositional data analysis, as stated by Aitchison (1986). In future work, the impact of Aitchison distance on t-SNE and the classification performance of compositional data should be studied with more rigorous mathematical theories.

In our data analysis we found that SVM outperformed LR and DT in many settings, which may be related to the fact that the classifier generalization ability varies for different types of data (Hastie et al., 2009; Oliveira et al., 2018), impacting the prediction accuracy. On the other hand, the optimization parameters often have a large impact on classification performance, and a reasonable and feasible tuning method is necessary. In our approach, a grid search with fivefold cross-validation is employed for tuning parameters, iterating over many possible parameter combinations to maximize the classification accuracy (ACC). Grid search is one of the most widely used techniques and allows us to have a transparent parameter selection (Huang et al., 2012). Moreover, considering comparable sequencing depth (about 30,000 tags) and sequencing quality (Q20 > 95%) in each sample, the difference in model performances for different datasets may be attributed to the following factors: (1) the ICPP data is intestinal microbiome which has a higher microbial load and more complex microbial composition relative to that in oropharynx (mean of Shannon index 2.078 in ICPP data vs. 1.831 in MP data); (2) the smaller sample size in ICPP data probably limit the performance of model.

In this study, we only considered LR, SVM, and DT, whereas other models such as neural networks may have better classification performance depending on the datasets. In addition, the same idea in our approach may be also applied to other manifold learning dimension reduction techniques, such as the unified manifold approximation and projection (UMAP) developed by McInnes and Healy (2018). As a preliminary study, we compared the model performances using UMAP based on Euclidean distance and Aitchison distance. The results were presented in Supplementary Table 3 for both microbiome datasets, showing that the use of Aitchison distance led to more accurate classification, similar to the proposed t-SNE based approach. More comprehensive and theoretical investigations on different dimension reduction methods will be conducted in future work.

The visualizations by t-SNE may be helpful for our understanding on the performance of the proposed approach. As shown in Figure 2A for the MP infection data, the control and case groups were well separated, and a satisfactory classification performance was expected at *dim* = 2 or 3. On the other hand, the t-SNE map at 2D for the ICPP data (Figure 2B) did not show a clear clustering pattern for the case and control groups which suggested that a relatively large value of *dim* would be needed to achieve a satisfactory classification performance.

## Data Availability Statement

The original contributions presented in the study are included in the article/Supplementary Material, further inquiries can be directed to the corresponding author/s.

## Author Contributions

XLX, DL, and XMX designed the research. XLX and ZX performed data analysis. XLX and ZY created the figures. XLX, ZX, and ZY wrote the manuscript. XMX and DL advised and were the senior supervisor of the project. All authors read and approved the submitted manuscript.

## Conflict of Interest

The authors declare that the research was conducted in the absence of any commercial or financial relationships that could be construed as a potential conflict of interest.

## Abbreviations

t-SNE: t-distributed stochastic neighborhood embedding
LR: logistic regression
SVM: support vector machine
DT: decision tree
ACC: the classification accuracy
nMCC: the normalized Matthews correlation coefficient
AUC: the area under the receiver operating characteristic curve
AUPR: the area under the precision-recall curve.
