# Supplementary material for: A t-SNE Based Classification Approach to Compositional Microbiome Data
Source: Front Genet. 2020 Dec 14;11:620143. doi: 10.3389/fgene.2020.620143 (PMC7767995; doi:10.3389/fgene.2020.620143)
Supplement: Supplementary file 1 [file Table_1.DOCX]

Supplementary Material

**Supplementary Table 1**. Running time (seconds) for both t-SNE with Aitchison distance and t-SNE with Euclidean distance.

|  | MP infection | | ICPP | |
| --- | --- | --- | --- | --- |
|  | ED | AD | ED | AD |
| time = | 20.02s | 20.11s | 8.80s | 9.00s |
