# Supplementary material for: A t-SNE Based Classification Approach to Compositional Microbiome Data
Source: Front Genet. 2020 Dec 14;11:620143. doi: 10.3389/fgene.2020.620143 (PMC7767995; doi:10.3389/fgene.2020.620143)
Supplement: Supplementary file 2 [file Table_2.DOCX]

Supplementary Material

**Supplementary Table 2**. The ratios (%) of improvement in the performance of the proposed method using Aitchison distance compared to that using Euclidean distance.

|  |  | MP infection | | | | ICPP | | | |
| --- | --- | --- | --- | --- | --- | --- | --- | --- | --- |
|  | *dim* = | 2 | 3 | 5 | 7 | 2 | 3 | 5 | 7 |
| ACC | LR | 24% | 9% | 8% | 9% | 25% | 17% | 13% | 13% |
|  | SVM | 17% | 10% | 12% | 13% | 20% | 21% | 27% | 28% |
|  | DT | 20% | 10% | 12% | 10% | 12% | 18% | 19% | 17% |
| nMCC | LR | 25% | 9% | 8% | 9% | 13% | 18% | 8% | 7% |
|  | SVM | 21% | 10% | 10% | 12% | 14% | 16% | 10% | 11% |
|  | DT | 23% | 13% | 13% | 13% | 8% | 18% | 4% | 7% |
| AUC | LR | 26% | 9% | 8% | 9% | 16% | 10% | 8% | 12% |
|  | SVM | 15% | 11% | 11% | 10% | 10% | 12% | 16% | 15% |
|  | DT | 23% | 9% | 15% | 15% | 2% | 6% | 5% | 7% |
| AUPR | LR | 33% | 14% | 13% | 10% | 8% | 4% | 3% | 4% |
|  | SVM | 35% | 16% | 14% | 14% | 8% | 6% | 3% | 4% |
|  | DT | 30% | 14% | 12% | 12% | 2% | 1% | 3% | 1% |

ACC: the classification accuracy; nMCC: the normalized Matthews correlation coefficient; AUC: the area under the receiver operating characteristic curve; AUPR: the area under the precision-recall curve; LR = logistic regression; SVM = support vector machine; DT = decision tree.
