# Supplementary material for: A t-SNE Based Classification Approach to Compositional Microbiome Data
Source: Front Genet. 2020 Dec 14;11:620143. doi: 10.3389/fgene.2020.620143 (PMC7767995; doi:10.3389/fgene.2020.620143)
Supplement: Supplementary file 3 [file Table_3.DOCX]

Supplementary Material

**Supplementary Table 3.** Classification performances of the UMAP based method on the test set.

|  |  |  | MP infection | ICPP |
| --- | --- | --- | --- | --- |
|  |  | *dim* = | 3 | 5 |
| ACC |  | LR | 0.83 | 0.68 |
|  | ED | SVM | 0.83 | 0.64 |
|  |  | DT | 0.81 | 0.64 |
|  |  | LR | 0.95 | 0.74 |
|  | AD | SVM | 0.97 | 0.80 |
|  |  | DT | 0.89 | 0.72 |
| nMCC |  | LR | 0.82 | 0.72 |
|  | ED | SVM | 0.81 | 0.71 |
|  |  | DT | 0.79 | 0.70 |
|  |  | LR | 0.95 | 0.73 |
|  | AD | SVM | 0.97 | 0.79 |
|  |  | DT | 0.88 | 0.71 |
| AUC |  | LR | 0.91 | 0.79 |
|  | ED | SVM | 0.91 | 0.76 |
|  |  | DT | 0.86 | 0.72 |
|  |  | LR | 1.00 | 0.84 |
|  | AD | SVM | 1.00 | 0.84 |
|  |  | DT | 0.91 | 0.73 |
| AUPR |  | LR | 0.95 | 0.80 |
|  | ED | SVM | 0.95 | 0.75 |
|  |  | DT | 0.89 | 0.73 |
|  |  | LR | 0.99 | 0.81 |
|  | AD | SVM | 0.99 | 0.80 |
|  |  | DT | 0.89 | 0.75 |

ACC: the classification accuracy; nMCC: the normalized Matthews correlation coefficient; AUC: the area under the receiver operating characteristic curve; AUPR: the area under the precision-recall curve; UMAP: the unified manifold approximation and projection; AD: the models using Aitchison distance; ED: the models using Euclidean distance; LR = logistic regression; SVM = support vector machine; DT = decision tree. Note: UMAP was implemented using the R package umap and was trained with the following parameter settings: n_components = 3 for MP infection data (= 5 for ICPP data), n_epochs = 500 for MP infection data (= 200 for ICPP data), alpha = 1. The rest of the unmentioned parameters uses the default setting in the R package.
